# Case report: Heart Mate III for systemic right ventricular support in a patient with hypoplastic left heart syndrome

**DOI:** 10.3389/fcvm.2022.1070314

**Published:** 2023-01-19

**Authors:** Maja Hanuna, Jelena Pabst von Ohain, Nikolaus Haas, Christoph S. Mueller, Robert Dalla-Pozza, Marcus Fischer, Frank Born, Christine Kamla, Andre Jakob, Christian Hagl, Jürgen Hörer, Sebastian G. Michel

**Affiliations:** ^1^Department of Cardiac Surgery, Ludwig Maximilian University of Munich, Munich, Germany; ^2^Division of Congenital and Pediatric Heart Surgery, Department of Cardiac Surgery, Ludwig Maximilian University of Munich, Munich, Germany; ^3^Department of Congenital and Pediatric Heart Surgery, German Heart Center Munich, Technical University of Munich, Munich, Germany; ^4^Department of Pediatric Cardiology and Intensive Care, Ludwig Maximilian University of Munich, Munich, Germany; ^5^Munich Heart Alliance, German Center for Cardiovascular Research, Munich, Germany

**Keywords:** failing Fontan, mechanical circulatory support, VAD—ventricular assist device, right ventricular (RV) failure, hypoplastic left heart syndrome, case report

## Abstract

Ventricular assist device implantation presents a possible bridge to heart transplantation for patients with failing Fontan physiology. However, evidence regarding outcome and possible pitfalls associated with the Fontan circulation is still insufficient. We describe the course of a 13-year-old male, who was born with hypoplastic left heart syndrome and underwent HeartMate III implantation due to refractory failure of the systemic right ventricle.

## 1. Introduction

The establishment of a Fontan circulation is essential to facilitate early survival of patients with functionally univentricular hearts but remains a palliative procedure nonetheless. Patients will eventually experience Fontan failure either due to reduced transpulmonary blood flow with or without increased pulmonary vascular resistance, resulting in reduced preload or failure (systolic or diastolic) of the systemic ventricle itself (1). Improved surgical and medical management led to a growing adolescent and adult patient cohort with Fontan physiology, ultimately qualifying for heart transplantation. As donor organ availability remains scarce, we are now observing an upturn in Fontan patients undergoing durable ventricular assist device (VAD) implantation (2–4). Herein, we describe the clinical course of a 13-year-old male with hypoplastic left heart syndrome (HLHS), who suffered from refractory systolic and diastolic failure of the systemic ventricle with unimpaired pulmonary blood flow and consequently received a HeartMate 3 (Abbott, Chicago, IL, USA) as a bridge to transplant.

## 2. Case description

**FIGURE 1:**
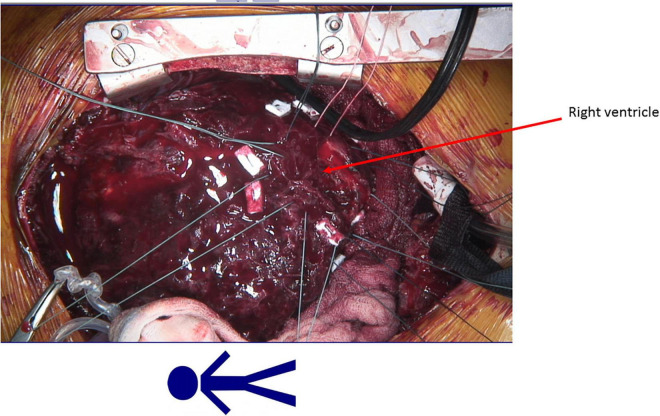
Eight interrupted pledgeted mattress sutures to fixate the Heart Mate 3 sewing ring in the right ventricle.

**FIGURE 2:**
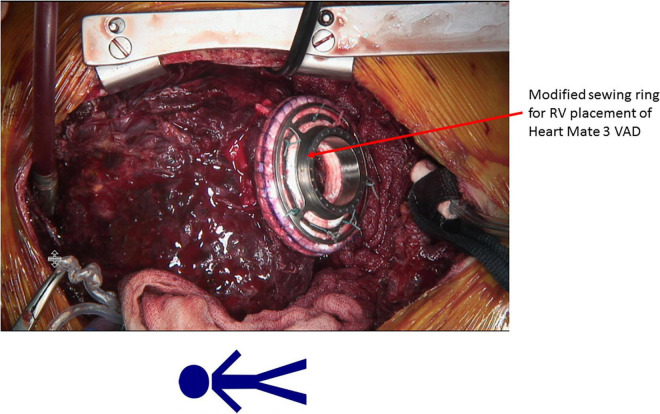
Modified sewing ring with four layers of felt pledget plates for right ventricular positioning of the Heart Mate 3.

**FIGURE 3:**
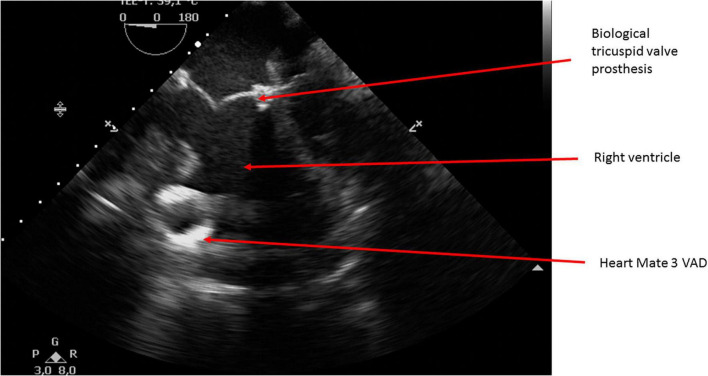
Transesophageal echocardiography showing pump position in the right ventricle at adequate distance from the tricuspid valve bioprosthesis.

**FIGURE 4:**
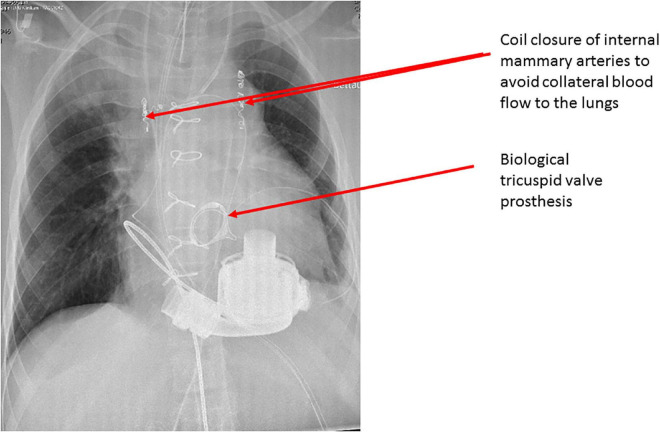
Postoperative chest x-ray showing adequate positioning of the Heart Mate 3 device in the right ventricle as well as the tricuspid valve bioprosthesis.

The patient was a 13-year-old male with HLHS who underwent Norwood procedure with a 5 mm Sano-shunt in the neonatal period. Bidirectional cavopulmonary anastomosis was performed at the age of 5 months, and the Fontan circulation was completed with an extracardiac non-fenestrated 18 mm Goretex conduit at the age of two years. At that time we also closed aorto-pulmonary collaterals to the right lung. Incipient reduction of exertional capacity was evident seven years after the total cavopulmonary anastomosis. By the age of 12, he suffered from symptoms of advanced heart failure (NYHA III; peak oxygen consumption of 11.8 ml/min/kg in cardiopulmonary stress testing). Echocardiography and magnetic resonance imaging revealed a high-grade tricuspid valve regurgitation due to severe annular dilation (60 mm) and a markedly reduced systemic ventricle ejection fraction of 36%. His last heart catheterization showed a mean pulmonary artery pressure of 16 mmHg, and a transpulmonary gradient of 4 mmHg with increased right ventricular end diastolic pressure of 12 mmHg, so elevated pulmonary vascular resistance was ruled out. There was no more significant collateral blood flow to the lungs at that time. He deteriorated and was admitted to our center in cardiogenic shock with hypotension and lactate acidosis peripheral veno-arterial extracorporeal life support (ECLS) was established using the femoral vessels. Tricuspid valve replacement (33 mm perimount) was performed on the 3rd day of ECLS support, which was continued postoperatively. Renal and hepatic functions were preserved while on ECLS support, but ECLS-weaning attempts were repeatedly unsuccessful. The decision was made to implant a HeartMate III (HM III) on the 9th day of ECLS treatment. The child was 45 kg and 165 cm which resulted in a body surface area of 1.43 m^2^. Surgical access was impaired due to extensive adhesions from previous procedures. ECLS was switched to cardiopulmonary bypass via the femoral vessels. The HM III was placed in the systemic—anatomically—right ventricle. Therefore, the sewing ring was enhanced with four layers of pledgets (Figures 1, 2) to reduce protrusion into the ventricular cavity as previously suggested for right sided VAD placement (5). Right-ventricular myocardial trabeculae, chords and parts of the papillary muscles of the tricuspid valve were resected to avoid inflow cannula obstruction. This was facilitated by the already implanted tricuspid valve bioprosthesis (Figures 3, 4). The modified sewing cuff was fixated with 8 interrupted pledgeted sutures. The outflow graft was anastomosed to the ascending aorta with 4-0 Prolene suture. Bypass time was 160 min. After weaning from cardiopulmonary bypass, the ventricular assist device (VAD) was gradually set at 5,800 rpm, reaching a flow of 4.5 L/min with a power consumption of 5.3 Watt. In the immediate postoperative period the patient was supported with inotropes and inhaled nitric oxide. The patient was extubated on the 4th postoperative day. A single run of renal replacement therapy was necessary on postoperative day 3. End-organ parameters were within normal range 3 weeks after VAD implantation. The postoperative course was complicated by bleeding events (hemothorax and GI-bleeding from esophageal varices). Mass transfusion was necessary during that period. After bleeding issues had been resolved, anticoagulation was started with heparine (target PTT = 60 s) and later was switched to Vitamin-K-antagonists Aspirine was not started until 6 weeks after surgery due to the GI bleeding. Additionally, impaired wound healing without evident infection could be observed at the driveline exit site. He was discharged from the hospital in good clinical condition on postoperative day 69. Logfiles remained unremarkable for low-flow or suction events. Evaluation for heart transplantation is currently ongoing.

## 3. Timeline


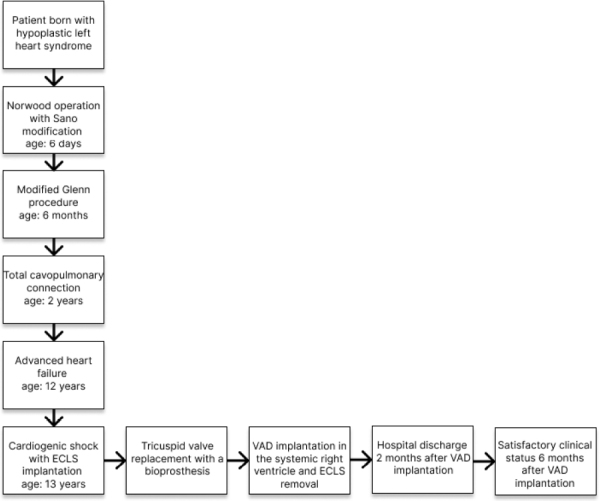


## 4. Discussion

Over the last two decades, durable mechanical circulatory support advanced to a solid second-line treatment after heart transplantation for pediatric patients suffering from end-stage heart failure. With growing experience in the pediatric community, improving survival data and decline in adverse events, patients with failing Fontan circulation are increasingly considered suitable for VAD implantation (2–4, 6). With the withdrawal of the HeartWare (Medtronic, Dublin, Ireland), the intracorporeal HM III and paracorporeal Berlin Heart EXCOR^®^ (Berlin Heart Medizintechnik GmbH, Berlin, Germany) remain the only options for pediatric durable support. We opted for the HM III as size was not an issue in our patient (body surface area 1.4 m^2^) and we aimed for hospital discharge during the expected long waiting time for heart transplantation. Additionally, intracorporeal flow devices are associated with the highest survival rates and fewer adverse events due to the lower thrombogenicity (6). However, patients with Fontan physiology represent a more challenging cohort and several additional pitfalls must be considered. Most importantly, low pulmonary vascular resistance is essential for unrestricted pulmonary blood flow and adequate VAD preload. This was the case in our patient, otherwise, a pulsatile sub-pulmonary assist device would have been necessary in addition to VAD support of the systemic ventricle. The details of this technique have been described previously (7). Numerous previous surgeries may result in relevant adhesions limiting surgical access, as presented in our case. Thus, alternative pump positions, modifications of the sewing cuff and generous resection of intraventricular material may be necessary to enable unobstructed pump-flow. Bleeding complications are not unexpected and therefore a well-established concept for the postoperative balance between early anticoagulation and bleeding complications (especially in Fontan-patients on ECLS-support prior to VAD implantation) is crucial. Last, special attention should be given to wound care. Reduced tissue integrity and interstitial edema caused by the elevated central venous pressure in patients with Fontan circulation could lead to impaired wound healing, which poses a greater risk for driveline infections. Despite additional challenges, our case indicates that HM III implantation is a feasible therapeutic option for patients with failure of the systemic ventricle and preserved pulmonary Fontan circulation.

## Data availability statement

The original contributions presented in this study are included in the article/supplementary material, further inquiries can be directed to the corresponding author.

## Ethics statement

Ethical review and approval was not required for the study on human participants in accordance with the local legislation and institutional requirements. Written informed consent to participate in this study was provided by the participants’ legal guardian/next of kin.

## Author contributions

MH, SM, JP, and JH wrote the first draft of the manuscript. All authors contributed to data analysis and data curation, contributed to manuscript revision, read, and approved the submitted version.
